# Selection of the best point and angle of lateral ventricle puncture according to DTI reconstruction of peripheral nerve fibers

**DOI:** 10.1097/MD.0000000000013095

**Published:** 2018-11-09

**Authors:** Sheng Zhong, Weihang Li, Bin Wang, Jiaxin Ren, Hui Li, Yingjing Zhao, Shanshan Jiang, Yuxiang Fan, Ye Cheng, Gang Zhao, Xinrui Liu, Rihua Jin

**Affiliations:** a Department of Neurosurgery, the First Hospital of Jilin University; b Clinical College, Jilin University; c Pharmacy College, Jilin University, Changchun; d Department of Neurosurgery, Xuanwu Hospital, Capital Medical University, Beijing, China.

**Keywords:** computed tomography, diffusion tensor imaging, lateral ventricle puncture, ventricle hemorrhage

## Abstract

This study aims to find accurate angles and depths of lateral ventricle puncture using diffusion tensor imaging (DTI) reconstruction, as well as to provide an optimized and alternative puncturing strategy.

A total of 90 computed tomography (CT) images and 30 CT images with DTI were analyzed. The measurements were performed on coronal, sagittal, and horizontal planes. Some distances and angles were measured to determine the best angle and penetration depth during the puncture process. Important landmarks of the lateral ventricle were also measured, and a comparison of the differences between 2 hemispheres was also assessed.

It showed that the vertical distance from the superior margin to inferior margin of the lateral ventricle was 22.2 ± 0.5 mm and the length was 124.1 ± 2.1 mm. In the frontal horn puncture approach, the penetration depth should be limited between 105.2 and 109.4 mm, the angle should be 71.6 ± 2.7°. During the occipital horn puncture approach, puncturing depth was from 90.7 to 111.4 mm, and angle was 15.3 ± 1.8°. Through the parietal lobe puncture approach, which was firstly brought out in this study, the puncturing length should be 124.4 to 130.2 mm and angle was 56.6 ± 2.0°.

The traditional recommended protocol of lateral ventricle puncture is not accurate, the refined lateral ventricle puncture protocol established in this study will reduce injury and remain function. A DTI imaging examination combining with nerve fibers reconstruction were strongly recommended before lateral ventricle puncture, which will help neurosurgeons to determine the best puncturing angles and depth.

## 1 Introduction

Lateral ventricles, 2 largest cavities of the ventricular system of the human brain which containing cerebrospinal fluid (CSF), are one of the most important parts of brain.^[1]^ Each lateral ventricle resembles a C-shaped structure from inferior horn in the temporal lobe, and pass through a body in the parietal lobe and frontal lobe. Once the lateral ventricle is damaged, it will cause some severe problems such as ventricle hemorrhage, which will lead to cerebrospinal fluid occlusion, hydrocephalus, ventricular expansion, increased intracranial pressure, brain herniation, brain stem, and hypothalamus damage. Besides, the ventricle hemorrhage will cause the hyperpyrexia, organ collapse, and lots of fatal complications. The hemorrhage of the lateral ventricle has become a major public health problem in recent years. It is well-accepted to eliminate the patients’ ventricle hemorrhage and relieve intracranial pressure by lateral ventricle puncture when hemorrhage occurs.^[2]^

Compared with craniotomy, the lateral ventricle puncture is minimally invasive, and maximally safe. Nevertheless, there are also some ventricle puncture failure reports, which will lead to many serious complications: infection, shunt vessel plug, intracranial hemorrhage, etc.^[3]^ Blames were put on the improper and inaccurate position of the puncturing points and angles, because this procedure mainly relies on the experience of neurosurgeons. Besides, the morphology of nerve fibers and lateral ventricle are quite different between individuals. Previous studies have already set up an established protocol of lateral ventricle puncture: puncture point was set as 10 cm superior to the nose root, 3 cm on the right side of the middle line, after infiltrating anesthesia and drilling skull, puncture was conducted on the frontal horn.^[4]^ However, the morphology of lateral ventricle in pathologic and physiologic conditions varies a lot. Additionally, nerve fibers between different races and ages have significant variations. Therefore, the previous established procedure is not accurate and it still needs to be improved. It is very necessary to conduct such an imaging study to determine the best points and angles to perform lateral ventricle puncture safely and effectively.

Diffusion tensor imaging (DTI) is a magnetic resonance imaging technique that enables the measurement of the restricted diffusion of water in nerve fibers in order to produce neural tract images, it provides useful as well as accurate structural information about nerve fibers.^[5]^ It also allows us to sketch detailed morphologic information of nerve fibers around lateral ventricle, which have never been reported before. Here, we conducted such a study that aimed to determine the most proper points and the best angles to perform lateral ventricle puncture by DTI and CT imaging, which could reduce the injury rapidly when puncturing. Through the comprehensive investigation, an innovative lateral ventricle puncture procedure via parietal lobe was firstly brought out. Besides, this procedure combined with other 2 traditional lateral ventricle puncture procedure were fully evaluated and compared to each others to elucidate the merits and disadvantages of each procedure. This study not only contributed to a better neuro-imaging understanding of the lateral ventricle and its peripheral structures, but also provided refined procedures to guide the operations.

## 2 Materials and methods

The DTI measurement protocol was performed at Department of Radiology, the First Hospital of Jilin University. This study was approved by the Ethics committee of the First Hospital of Jilin University. All participants were given written informed consent. We enrolled 30 healthy adult participants from December 2016 to May 2017 who are more than 18 years old and volunteered to participate in the study, 30 CT and 30 DTI were acquired from the same adults for study. In addition, other retrospective imaging data of 90 healthy adults were also included in the present study. Participants were considered only if exhibited no symptoms of neurology. The exclusion criteria are pathologic or traumatic effects in their cerebral DTI image. The participants in this study including 57 males and 63 females aged from 22 to 87 years, with an average age of 52.5 years. DTI data were processed by DSI studio software to reconstruct the parameter of fractional anisotropy (FA), volume ratio (VR), relative anisotropy (RA), apparent diffusion coefficient (ADC), and the color FA map. The bilateral data were measured, and a comparison of the differences between 2 hemispheres was also assessed.

### 2.1 CT imaging method

**Figure 1:**
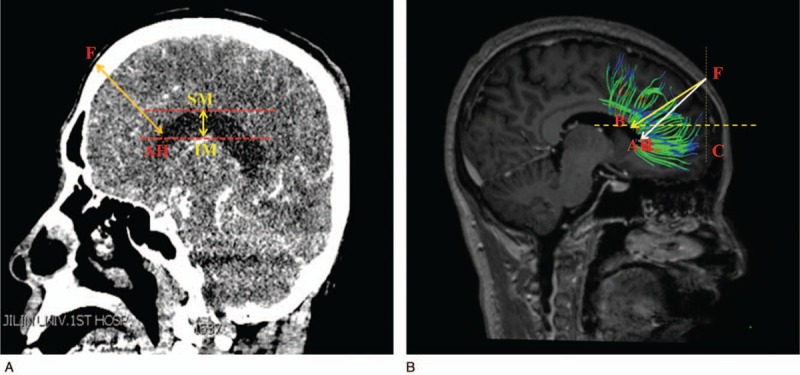
(A) The data and the puncture point from frontal lobe in computed tomography image. (B) The puncture point and angle in diffusion tensor image.

**Figure 2:**
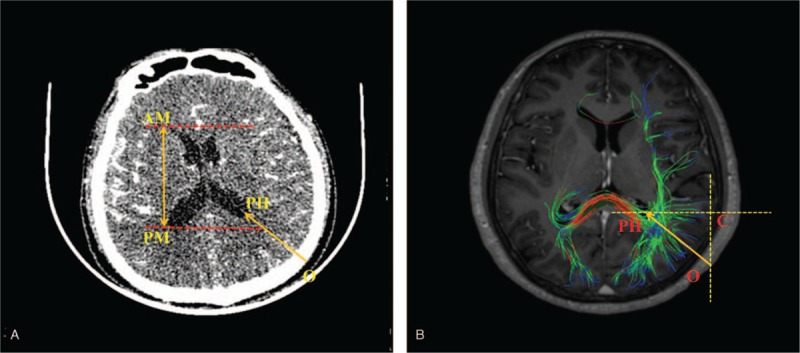
(A) The data and the puncture point from the occipital lobe in computed tomography image. (B) The puncture point and angle in diffusion tensor image.

**Figure 3:**
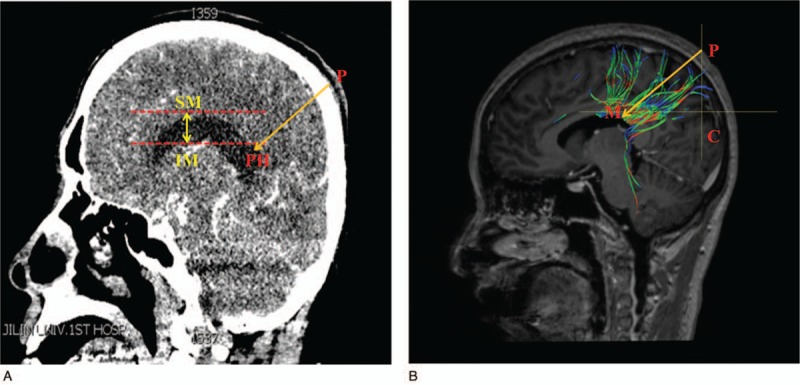
(A) The data and an innovative puncture method from the parietal lobe in computed tomography image. (B) The new innovative method's puncture point and angle in diffusion tensor image.

All the CT imaging data were taken by a CT machine (GE Sigma HDxt; GE, Fairfield, CT) in Department of Radiology, the First Hospital of Jilin University. Then, the imaging data were reconstructed using the volume-rendering system to conduct a measurement. The measurements were performed on the coronal, sagittal, and axial planes after three-dimension reconstruction in computer, respectively. After all the anatomical structures were identified, the sagittal plane was selected to make the measurements (as shown in Fig. 1A). The shortest distance (FAH) from the frontal puncture point (F) to anterior horn of the lateral ventricle (AH) was measured, and the vertical distance of the lateral ventricle was also measured. The vertical distance of the lateral ventricle was defined as the distance from inferior margin (IM) to superior margin (SM) of the lateral ventricle in sagittal plane. Then, the horizontal plane of the samples was selected (as shown in Fig. 2A), we also measured the shortest distance (OPH) from the occipital puncture point (O) to the posterior horn of the lateral ventricle (PH) as well as the length of the lateral ventricle, the length of the lateral ventricle was defined as the distance from anterior margin (AM) to posterior margin (PM) of the lateral ventricle in axial plane. What's more, the other sagittal plane of the samples was selected as an innovative method brought out in this study, the puncture line was chosen by parallel to the most of never fiber bundles (PM) (as shown in Fig. 3A), we measured the shortest distance PPH from the parietal puncture point (P) to the posterior horn of the lateral ventricle (PH) as well as the distance of the lateral ventricle from IM to SM of the lateral ventricle in sagittal plane.

### 2.2 DTI method

The DTI data acquisition: Using the Siemens 1.5 TMR scanner, gradient field 40 mt/M, switching rate of 200 mT/M/ms. The axial position was conducted by using the diffusion sensitive single-induced echo plane imaging sequence, the scanning parameters are: TR = 8100 milliseconds, TE = 106  milliseconds, FOV = 230 mm × 230 mm, 128 by 128 matrix, 3 mm layer thick, no interval, total 40-story image. By using 2 dispersion weights, *b* value is 0 and 1000 s/mm^2^, respectively. Diffusion sensitive gradient is applied to the 6 isotropic directions, respectively. Total collection time is 7 minutes and 30 seconds.

**Figure 4:**
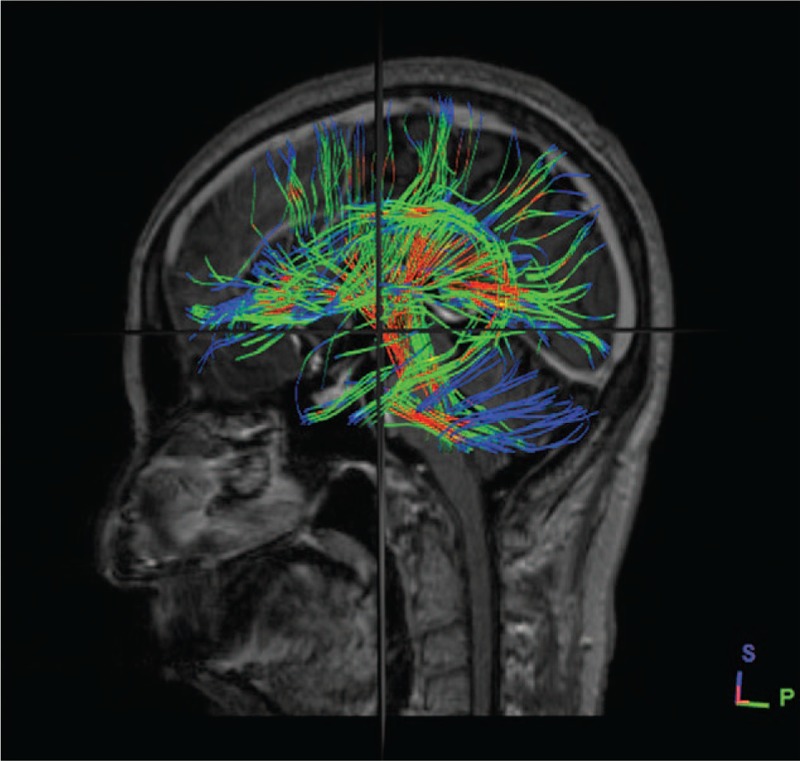
Whole fiber bundles of brain were reconstructed by DTT technique.

Image processing: DSI studio was used to analyze the image and to reconstruct the FA, VR, RA, ADC, and color FA map. By comparing and analyzing the section images, FA and color FA map, the location and form of the white matter fiber bundle as well as the lateral ventricle were identified and measured. After all the anatomical structures were identified, the reconstructed whole brain never fibers was displayed (as shown in Fig. 4), the axial parameters S, P, and L noted superior, posterior and left, respectively. The sagittal plane of the samples which reconstructed partial never fibers near the frontal lobe was selected (as shown in Fig. 1 B), we measured the shortest distance FAH from the frontal puncture point (F) to the anterior horn of the lateral ventricle (AH), the distance FB from the puncture point (F) to the point B which was determined by the direction parallel to the fiber bundles. In this way, FB was parallel to the shape of fiber bundles. FB is the refined puncturing angle that was firstly brought out in this study, whereas FAH was the traditional puncturing route. The vertical distance of the lateral ventricle from IM to SM of the lateral ventricle in sagittal plane and ∠BFC, the angle between line FB and coronal plane (C) was also measured. The horizontal plane of the samples which reconstructed partial never fibers near the occipital lobe was selected (as shown in Fig. 2B), we measured the distance (OPH) from the occipital puncture point (O) to the posterior horn of the lateral ventricle (PH) as well as the length of the lateral ventricle from AM to PM of the lateral ventricle in axial plane. ∠OPC, the angle between line OPH and coronal plane (C) was also assessed. The sagittal plane of the samples which reconstructed partial never fibers near the parietal lobe was selected (as shown in Fig. 3B), we measured the distance PM from the parietal puncture point (P) to the point which was determined by parallel to the fiber bundles (M). Therefore, PM was parallel to the shape of fiber bundles. The vertical distance of the lateral ventricle from IM to SM of the lateral ventricle in sagittal plane and ∠MPC, the angle between the line MP and coronal plane (C), were measured. Measurement data of the length and height of the lateral ventricle can help neurosurgeons to determine the morphology of the lateral ventricle as well as surrounding never fiber bundles easily and accurately, never fiber bundles can be identified with the help of lateral ventricle morphology.

### 2.3 Statistical analysis

All the statistics were entered into SPSS 18.0 (SPSS Inc, Chicago, IL) for analysis. The measurements were presented as mean ± standard deviation. We had normality tests for all the data. Differences between the 2 hemispheres were tested by an independent-sample *t* test. *P* < .05 noted that there was a statistical significance between 2 hemispheres.

## 3 Results

There was no significant difference between 2 hemispheres (*P* > .05). In addition, no significant differences were found between men and women (*P* > .05). Three lateral ventricle puncture procedures were fully investigated and measured as follows.

### 3.1 Lateral ventricle puncture from the frontal horn approach

The puncture point was selected as 10 cm superior to the nose root, 3 cm on the right side of the middle line, infiltrating anesthesia, and drilling skull. Point F in Figure 1 was considered as the puncture point. CT measurement results showed that the shortest distance between the puncture point (F) and anterior horn of the lateral ventricle (AH) was 94.7 mm, and the vertical distance of the lateral ventricle was 22.3 ± 0.3 mm. Then, DSI studio was used to reconstruct the important fiber bundles which were surrounding the lateral ventricle. The distance and angles were also measured in DTI image. DTI measurement results showed that the shortest distance between the puncture point (F) and anterior horn of the lateral ventricle (AH) was 94.6 mm, and the vertical distance of the lateral ventricle was 22.2 ± 0.5 mm. Results showed that the shortest distances and the vertical distance of the lateral ventricle in the CT measurement and DTI measurement were roughly the same. In this study, it also showed that the traditional puncture route tended to cause more damage and injury, because the traditional puncture route is not parallel to the shape of the lateral ventricle. Therefore, a refined frontal puncture procedure was firstly brought out by us, shown as the direction of FB in Figure 1B. The line FB was parallel to the most of fiber bundles, in this way, puncture process will cause less injury and damage though the distance FB was 105.2 ± 2.3 mm, which is a little longer than FA (traditional puncture route). Additionally, the DTI image can also be used to demonstrate the fiber bundle's position and its angles. The puncturing angle ∠BFC in the refined frontal puncturing process ought to be 71.6° in the sagittal plane. The data of landmarks location implied that puncturing depth should be limited from 105.2 to 109.4 mm and the puncturing angle was 71.6 ± 2.7°, exceeding this value maybe damage other important structures or nerve fibers. All data of the CT and DTI measurements are shown in Table 1.

**Table 1:**
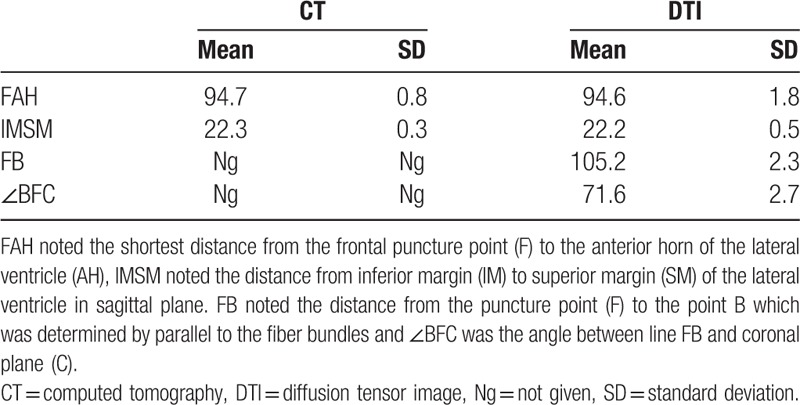
Measured values of FAH, IMSM, FB, and BFC from CT and DTI (mm and °).

### 3.2 Lateral ventricle puncture from the occipital horn approach

The puncture point was located as 4 to 7 cm superior to the external occipital protuberance and 3 cm next to the middle line. Point “O” in Figure 2 was considered as the puncture point. Initially, CT image was used to measure the shortest distance between the puncture point (O) and the posterior horn of the lateral ventricle (PH), the shortest distance was 89.7 mm and the length of the lateral ventricle, the distance between the anterior horn and posterior horn of the lateral ventricle, was 124.6 ± 1.5 mm. Then, DTI was also used to conducted measurements. DTI measurements showed that the shortest puncturing distance was 90.7 mm and the length of the lateral ventricle was 124.1 ± 2.1 mm. In addition, DTI showed that the nerve fibers surrounding the posterior horn of the lateral ventricle was sophisticated and contorted, which demonstrated that posterior horn puncture was not an ideal procedure, and it can cause a lot of complications related to vision. OPH was selected as the puncturing route in the occipital horn puncture approach in Figure 2B, which was parallel to the most of fiber bundles surrounding the lateral ventricle. The distance OPH was measured as 90.7 ± 1.3 mm. The angle ∠OPC between the fiber bundles and coronal plane was 15.3° in the horizontal plane. The measurement data implied that puncturing depth should be limited from 90.7 to 111.4 mm, and puncturing angle was 15.3 ± 1.8°, exceeding this value the puncturing maybe not reach the lateral ventricle as well as damage some other important structures. The data of all CT and DTI landmarks measured in this method are shown in Table 2.

**Table 2:**
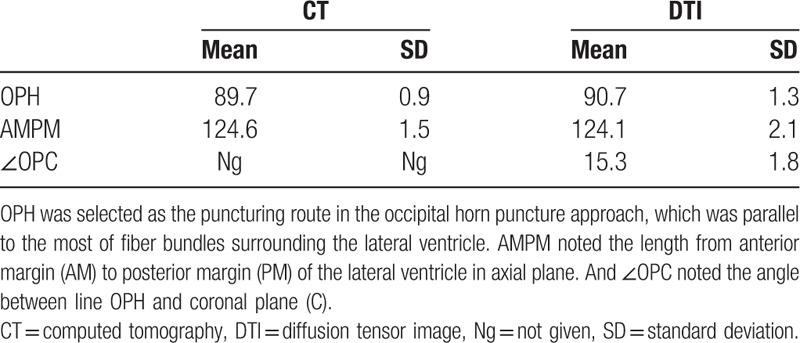
Measured values of OPH, AMPM, and OPC from CT and DTI (mm and °).

### 3.3 Lateral ventricle puncture from the parietal lobe approach

This study was an innovative method that firstly developed in this study by DTI technology. The puncture point was selected as 35.5 cm superior to the nasal root and 2 cm next to the midline. Point P in Figure 3 was considered as the puncture point. First of all, the CT image was still used to conduct basic anatomical measurements, the shortest distance between puncture point (P) and the posterior horn of the lateral ventricle (PH) was 124.4 mm and the vertical distance of the lateral ventricle was 22.3 ± 0.3 mm. Secondly, the DTI technology was used to measure the puncturing angles and depth more accurately. Results showed that there was no significant difference between CT measurement and DTI measurement. The line PM was selected as puncture route which was parallel to the most of fiber bundles which were surrounding the lateral ventricle, and the distance PM was measured as 127.4 ± 2.3 mm. DTI results also showed that the angle ∠MPC which was between the line PM and coronal plane (C) was 56.6°. The measurement data implied that puncturing length should be limited between 124.4 and 130.2 mm and angle was 56.6 ± 2.0°, exceeding this value it might cause severe damage and injury for patients. The detailed measurements of CT and DTI are shown in Table 3.

**Table 3:**
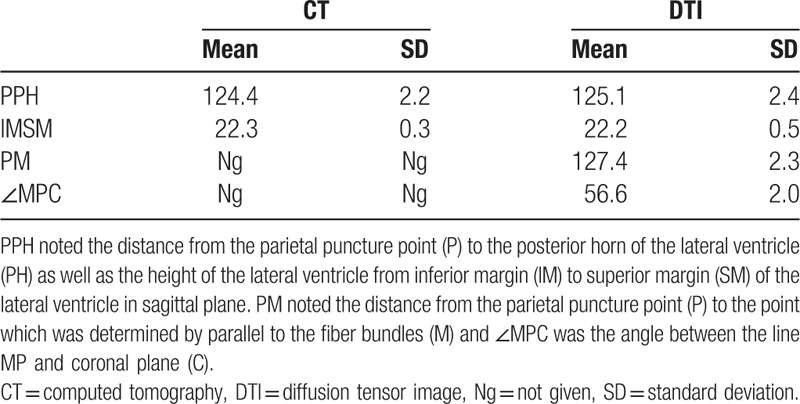
Measured values of PPH, IMSM, PM, and MPC in CT and DTI.

## 4 Discussion

Lateral ventricle hemorrhage is a common but vital hemorrhagic cerebrovascular disease. Some severe complications such as hematoma and different types of cerebrospinal fluid circulation disorder may occur due to various amount of hemorrhage. Since there are plenty of supplying arteries around this region, lateral ventricle hemorrhage tend to cause obstructive hydrocephalus, which will lead to an increase of intracranial pressure, ultimately forming cerebral hernia.^[6]^ Besides, the hemorrhage breaking into the lateral ventricle will alter the pressure in thalamus and brain stem, which would finally lead to endocrine dysfunction, abnormal circulation as well as respiratory systems malfunction and some other complications. Lateral ventricle puncture approach is a very effective way to treat lateral ventricle hemorrhage or obstructive hydrocephalus, which is well-accepted worldwide.

Traditional management in the puncture process mostly depends on operator's experience because of the lack of advanced image data and accurate locating devises.^[7]^ Therefore, it is difficult to determine a precise puncture point simply relies on CT image, operators usually tend to apply the shortest puncture procedure in clinical practice. However, there are plenty of failure cases and complications that occur every year due to inaccurate location and misunderstanding regarding to this procedure. The complications include cognition impairment, cerebral visual injury, hemorrhage, etc.^[8]^ With the rapid development of modern science and technology, this situation needs to be improved urgently. Therefore, DTI technology combined with CT was used in this study to determine the best puncturing depth and angles in different procedures, additionally the advantages, disadvantages, and indications of each approach was also fully elucidated.

The DTI technology enabled us to observe the location, morphology, and the positional relationships of the fibers as well as the lateral ventricle clearly and accurately. Magnetic resonance imaging (MRI) 3D-rendering tools were used to analyze the T1-weighted images and DTI in DSI studio. By reconstructing the fiber bundles, the spatial relationships between fiber bundles and lateral ventricle were displayed. After calculating the angles between the sagittal, coronal, axial plane, and fiber bundles, the best angles to perform lateral ventricle puncture were determined

### 4.1 Lateral ventricle puncture from the frontal horn approach

With the help of DTI technology, the puncturing depth and angles were determined by reconstructing never fiber bundles. In the reconstruction data and images, it showed that the nerve fiber of the frontal lobe is straight and regular, and there are only a little variations in the traveling course of the nerve fiber among individuals. Therefore, this part is an ideal region to perform lateral ventricles puncture. However, reconstruction results also showed that the traditional operation process (the direction of FAH, shown in Fig. 1) cross the main direction of the most nerve fiber bundles in this region, which demonstrated that the traditional operation process will cause more damage regarding to brain. Due to the important function of frontal lobe, the puncture procedure was refined in this study to reduce the damage and injury of brain. The puncturing angle should be changed to 71.6 ± 2.7°, and the puncturing depth altered to 105.2 to 109.4 mm accordingly. Though the puncture depth increased a little, it indeed reduced the damage of brain. With the help of DTI technology, it could be seen clearly that the traditional operation process FAH was much different from the refined puncture direction of FB, it would lead to much more damage and cause cognitive dysfunction. However, these complications could be avoided in the refined approach by setting the puncturing direction parallel to most of the nerve fiber bundles. Accordingly, the damage of fiber bundles reduced significantly. Additionally, the result also proved that by the reconstruction of the never fiber bundles, the shape of fibers near the later ventricle could be seen clearly, in this way, it provided a solid reference and a better comprehension for the puncture operation. An individual MRI and DTI examination was recommended before puncture to achieve better therapeutic effects.

### 4.2 Lateral ventricle puncture from the occipital horn approach

This approach is the standard procedure in the cerebral ventricular shunt process, with the help of DTI technology combined with CT technology, we found that many refinement needs to be improved regarding to this approach. By reconstructing the never fiber surrounding the lateral ventricle, it showed that the nerve fiber of this region contorted with each other, fiber bundles of this region is messy because of Gratiolet bundles and other nerve fibers, so it is difficult to avoid all the nerve fibers. Besides, tractus opticus travelled across this region, many complications related to the vision sometimes occurred after treatment. Therefore, an ideal approach should avoid all the important fiber and nerve tracts. According to the DTI result, puncturing depth should be limited from 90.7 to 111.4 mm, and puncturing angle was 15.3 ± 1.8° in the occipital lateral ventricle approach. With this approach, the puncturing needle will cause the least damage and reach the posterior horn in a safe way. Compared with other 2 approaches, this approach tends to cause more complications.

### 4.3 Lateral ventricle puncture from the parietal lobe approach

This method was a new innovative method which is firstly brought out in this study by DTI reconstruction. The puncture point was set posterior to postcentral gyrus. Traditional concepts believed that the longer puncture depth it had, the severe damage it would cause. However, our study demonstrated it was apparently a popular misunderstanding. Based on the DTI/diffusion tensor tractography (DTT) reconstruction, we could draw a firm conclusion that the key point causing complications and postsurgery damage was not the puncturing depth but the puncturing angles. If the angle is improper or inaccurate, the fiber bundles can be damaged no matter how deep the penetration is, resulting in character disorder or visual impairment. Additionally, nerve fiber bundle of this region is relatively regular and trim, the variation of fibers in this region is not significant. Besides, this region dominate the feeling of a person, compared with the character disorder or visual impairment caused by the damage of frontal or occipital lobe, paresthesia may be the lowest cost which could be accept by most people. With the anatomical data provided in this study, this innovative procedure is much easier to be conducted and it tends to cause less damage compared with the other 2 traditional procedures.

In this study, we provided not only a new innovative technology but also a completely new puncture strategy which had never been used before. The DTI technology could not only observe the tracking fibers of a brain but also determine the best puncturing angles and depth in different operations. In addition, measurements data in this study are also easy to be transformed in other coordinate systems which are different from ours. Sometimes the damage of the fiber bundles is inevitable, but the damage extent can be reduced rapidly by choosing a best puncture point and a proper angle. When a neurosurgeon conducted lateral ventricle puncture, it is easy to determine the best puncturing angles and depth by a protractor and a ruler with the help of measurement in this study.^[9]^ This study not only provides neurosurgeons a better comprehension regarding to positional relationship of the lateral ventricle and peripheral tracts, but also provide a detailed guideline for lateral ventricle puncture, which would reduce intraoperative or postoperative accidents rapidly. In addition, DTI technology had broad application in neurosurgery. A DTI imaging examination combining with nerve fibers reconstruction was strongly recommended before lateral ventricle puncture, which will help neurosurgeons to determine the best puncturing angles and depth for individuals.

Though this study is concluded by elaborate design and precise measurements, we still have to admit that the sample of this study is still limited, further studies need to be conducted in the future to confirm our conclusions.

## Author contributions

Zhong: Manuscript editing. Li: Manuscript writing/editing. Wang: Protocol development. Ren: Data collection. Li: Data management. Zhao: Data collection. Jiang: Data analysis. Liu: Data collection. Fan: Data analysis. Cheng: Project development. Zhao: Data management.

**Conceptualization:** Sheng Zhong, Weihang Li, Yuxiang Fan.

**Data curation:** Sheng Zhong.

**Formal analysis:** Bin Wang.

**Funding acquisition:** Weihang Li, Bin Wang, Gang Zhao.

**Investigation:** Jiaxin Ren, Ye Cheng.

**Methodology:** Jiaxin Ren.

**Project administration:** Yuxiang Fan.

**Resources:** Hui Li.

**Software:** Hui Li, Ye Cheng.

**Supervision:** Xinrui Liu.

**Validation:** Shanshan Jiang, Gang Zhao.

**Visualization:** Shanshan Jiang, Xinrui Liu.

**Writing – original draft:** Yingjing Zhao.

**Writing – review & editing:** Yingjing Zhao.
